# Temperature and Soil Moisture Stress Modulate the Host Defense Response in Chickpea During Dry Root Rot Incidence

**DOI:** 10.3389/fpls.2021.653265

**Published:** 2021-06-04

**Authors:** U. S. Sharath Chandran, Avijit Tarafdar, H. S. Mahesha, Mamta Sharma

**Affiliations:** ^1^Legumes Pathology, Integrated Crop Management, International Crops Research Institute for the Semi-Arid Tropics, Patancheru, India; ^2^Crop Improvement Division, ICAR-Indian Grassland and Fodder Research Institute, Jhansi, India

**Keywords:** *Rhizoctonia bataticola*, dry root rot, disease susceptibility index, combined stress, differential gene expression

## Abstract

Dry root rot caused by the necrotrophic phytopathogenic fungus *Rhizoctonia bataticola* is an emerging threat to chickpea production in India. In the near future, the expected increase in average temperature and inconsistent rainfall patterns resultant of changing climatic scenarios are strongly believed to exacerbate the disease to epidemic proportions. The present study aims to quantify the collective role of temperature and soil moisture content (SMC) on disease progression in chickpea under controlled environmental conditions. In our study, we could find that both temperature and soil moisture played a decisive role in influencing the dry root rot disease scenario. As per the disease susceptibility index (DSI), a combination of high temperature (35°C) and low SMC (60%) was found to elicit the highest disease susceptibility in chickpea. High pathogen colonization was realized in chickpea root tissue at all time-points irrespective of genotype, temperature, and SMC. Interestingly, this was in contrast to the DSI where no visible symptoms were recorded in the roots or foliage during the initial time-points. For each time-point, the colonization was slightly higher at 35°C than 25°C, while the same did not vary significantly with respect to SMC. Furthermore, the differential expression study revealed the involvement of host defense-related genes like endochitinase and PR-3-type chitinase (*CHI* III) genes in delaying the dry root rot (DRR) disease progression in chickpea. Such genes were found to be highly active during the early stages of infection especially under low SMC.

## Introduction

Chickpea (*Cicer arietinum* L.) is an essential crop for semi-arid tropics having the niche for cultivation in several developed and developing countries (Imtiaz et al., 2011). Globally, chickpea is cultivated in an area of 14.56 m ha, having an annual production of 14.78 m t. India has 9.54 m ha of the area under chickpea cultivation and contributes nearly 61.23% to the world’s total chickpea production (FAOSTAT, 2017).

Both biotic (Ghosh et al., 2016, 2017) and abiotic stresses (Palit et al., 2020) are known to impede chickpea production leading to reduced yields; among which, the former tends to pose a much larger constraint in the advent of rapidly changing climatic scenarios. Fusarium wilt has remained the major destructive disease in chickpea cultivation over the past many decades (Sharma et al., 2016). But, a major shift in the occurrence and spread of soil-borne diseases has been observed in chickpea over the past decade, in addition to the appearance of new and emerging diseases (Sharma and Ghosh, 2017; Chobe et al., 2020).

Dry root rot (DRR) of chickpea caused by *Rhizoctonia bataticola* (Taubenhaus) E. J. Butler [Syn: *Macrophomina phaseolina* (Tassi) Goidanich], a necrotrophic soil-inhabiting pathogen, has become an emerging threat to chickpea production in the recent decade due to climate change (Sharma et al., 2015; Chobe et al., 2019). Disease surveys conducted in chickpea-growing areas of central and southern India in the past and recent years have reported the incidence of DRR to vary from 5 to 35%, wherein Madhya Pradesh, Karnataka, and Andhra Pradesh were reported to be the emerging hotspots for DRR in chickpea (Ghosh et al., 2013; ICRISAT Happenings, 2019). The incidence of DRR is high when chickpea is under moisture deficit conditions with infection and disease progression optimum at higher soil temperatures of 35°C and soil moisture content (SMC) ≤60% (Sharma and Pande, 2013). In the near future, an increase in average temperature and inconsistent rainfall patterns resultant of changing climatic scenarios are strongly believed to exacerbate DRR of chickpea to epidemic proportions. Several previous reports suggest the role of soil moisture and temperature in DRR incidence on chickpea (Sharma and Pande, 2013; Sharma et al., 2015; Ghosh et al., 2017), but scanty information is available on the molecular responses exhibited during host-pathogen interaction.

A large number of studies reveal that plants respond to combined stresses differently from how they do under individual stress by activating a definite set of gene expression relating to the exact environmental conditions encountered. The presence of abiotic stress generally does not impose any additive effect but rather reduces or enhances the susceptibility of the plants toward the biotic pest or pathogen and vice versa (Sharma and Ghosh, 2017; Tarafdar et al., 2018). The low soil moisture condition (SMC) in a combined or multiple-stress scenario could elicit either a positive reaction or a negative one in the plant (Graham and Vance, 2003); like in several instances, plants have shown to become more susceptible to bacterial diseases under low SMC (Mohr and Cahill, 2003; Choi et al., 2013). Conversely, other studies do confirm low-moisture stress being able to improve the defense response of plants against such biotic stress elements (Ramegowda et al., 2013; Hatmi et al., 2015; Sinha et al., 2016). These may depend on the synergistic or antagonistic interaction of different components of drought and their corresponding defense signaling cascades through kinases and transcription factors (Kissoudis et al., 2014) influencing the plant’s tolerance against drought or pathogen. Similarly, the influence of temperature on disease response against phytopathogens has also been shown by various researchers. The molecular responses of several crops against phytopathogens in relation to soil moisture stress have been reported (Choi et al., 2013; Ramegowda et al., 2013; Hatmi et al., 2015; Tarafdar et al., 2018); however, only a few pertain to soil-borne pathogens.

Elements of climatic change are one of the key factors influencing the epidemiology of plant diseases (Graham and Vance, 2003; Zhao and Running, 2010; Sharma et al., 2019), as the life cycle of phytopathogens is heavily dependent on weather components such as temperature, rainfall, soil moisture, humidity, and greenhouse gases (IPPC, 2007; Sharma et al., 2015). Thus, to have a clear understanding of the host–pathogen interaction at different planes, viz., cellular, physiological, and molecular levels, it is imperative that we study plant diseases in a combined stress scenario having the interaction of both biotic and abiotic stresses, rather than its individual counterparts. Recently, Tarafdar et al. (2018) reported varied responses of the host pathogenesis-related (PR) genes in chickpea and pathogenicity-causing genes of *Sclerotium rolfsii* during collar rot development under different SMC. The present study was therefore aimed to assess and quantify the collective role of temperature and soil moisture on DRR progression in chickpea under simulated environmental conditions. Examining the above scenario from a molecular perspective is also crucial in this regard, as it would help device better control strategies against this disease. Keeping in mind the limited information presently available concerning DRR of chickpea, we have attempted to study the differences in the net impact of combined stress at the physiological as well as molecular level through differential gene expression studies in a compatible chickpea × *R. bataticola* pathosystem.

## Materials and Methods

### Plant Material

Two chickpea genotypes, viz., BG 212 and JG 11, were used for the present study. BG 212 is a highly susceptible genotype to DRR and is frequently used as a susceptible check during DRR screening and resistance breeding programs (Sharma and Pande, 2013). JG 11 is an early-maturing, bold-seeded, wilt-resistant, and commercially grown genotype.

### Fungal Isolate, Mass Multiplication, and Sick Soil Development

A highly pathogenic *R. bataticola* isolate, *Rb*6, was used throughout the study. For mass multiplication, *Rb6* was grown on potato dextrose agar (PDA) in Petri plates and incubated at 25 ± 1°C for 3–4 days with a 12-h photoperiod. Mycelial plugs cut from the periphery of an actively growing *Rb6* culture was used to inoculate sand-maize media and incubated at the temperature as before. As *R. bataticola* starts colonizing the media, it was thoroughly shaken every 2–3 days to break any clumps and ensure a uniform spread of inoculum. A 15-day-old, mass-multiplied inoculum was mixed with sterilized black soil at 50 g inoculum/kg of soil for the production of sick soil (Pande et al., 2012). Such soils were mixed thoroughly to confirm homogeneity and filled into 6-inch pots at 2 kg soil/pot. Sick soils were recurrently sown with susceptible variety BG 212 for two–three times until >90% disease incidence was recorded in susceptible genotypes. Each time, the healthy plants were rouged out while the infected plants chopped and incorporated into the soil. This sick soil was subsequently used in conducting the experiment.

Seeds of BG 212 and JG 11 were surface sterilized using 5% sodium hypochlorite for 1–2 min and washed twice thoroughly using sterilized de-ionized water. For both varieties, 10 seeds per pot (2 kg black soil/pot) were sown, adequately watered, and shifted to plant growth chambers (PGC). Pots filled with sick soil were referred to as inoculated soil, while pots with autoclaved black soil served as control and henceforth will be referred to as un-inoculated soil.

To study the host–pathogen (chickpea × *R. bataticola*) interaction under simulated environmental conditions, the experiment was undertaken in specialized PGC of Conviron in two scenarios. Here, the gradients for the climatic parameters (Supplementary Table 1), viz., temperature, relative humidity, and light intensity, were set in two PGC separately. Efforts were taken to establish the gradients such that one represents a cool scenario (PGC 1) with the peak temperature reaching a maximum of 25°C, whereas the second represents a warm scenario (PGC 2) with the peak temperature reaching a maximum of 35°C. The pots used for the study are of small 6-inch sizes; hence, we assume that any differences between the temperatures in the growth chamber and that of the potted soil to be small or negligible. Since the soil temperatures in field conditions are highly variable and difficult to mimic in controlled conditions, we have not accounted it in our study. To study the effect of SMC on infection and disease progression, the treatments under both scenarios were further divided into two sets of 60 and 80% SMC, respectively. The moisture regimes reflected poorly irrigated and well-irrigated field conditions, respectively.

Details of the entire experimental setup providing information on the combinations of temperature, SMC, and pathogen used during the study are given in Table 1. The experiment was kept in a completely randomized design (CRD) with all the treatments maintained as triplicates. A threefold number of pots was also kept to facilitate destructive sampling and subsequent analysis.

**TABLE 1:** Details of experimental setup and summary of observation.

Temperature (°C)	SMC (%)	Pathogen	Remarks/observations*
25	60	Sick soil	i. Low-to-moderate disease susceptibility ii. Plants under biotic + low soil moisture stress iii. Considered for gene expression studies
		Non-inoculated	i. No disease recorded ii. Plants under low soil moisture stress iii. Not considered for gene expression studies
25	80	Sick soil	i. Least disease susceptibility ii. Plants under biotic + least abiotic stress iii. Considered for gene expression studies
		Non-inoculated	i. No disease recorded ii. No biotic and abiotic stress available to plant iii. Optimum conditions for plant growth iv. Root tissues at 14 DAS was taken as control to normalize the gene expression profiles of plant defense-related genes
35	60	Sick soil	i. Highest disease susceptibility ii. Plants under very biotic + high abiotic stress iii. Optimum conditions for DRR development iii. Considered for gene expression studies
		Non-inoculated	i. No disease recorded ii. Plants under high abiotic stress iii. Not considered for gene expression studies
35	80	Sick soil	i. Low-to-moderate disease susceptibility ii. Plants under biotic + high-temperature stress iii. Considered for gene expression studies
		Non-inoculated	i. No disease recorded ii. Plants under high-temperature stress iii. Not considered for gene expression studies

*Observations for disease severity were taken at 28 DAS.

### Soil Moisture Stress Imposition, Sampling, and Observations

All pots were watered regularly for the first 8–10 days at 80% SMC to ensure proper germination and seedling emergence. Soil moisture stress imposition on the plants was initiated from 14 days after sowing (DAS). The SMC was determined using the gravimetric method on an oven-dry basis, where the pots were regularly weighed and any moisture deficit was made up by the addition of de-ionized water (Sharma et al., 2015). The first sampling was conducted at 14 DAS. Subsequently, this was accompanied by two more samplings with an interval of 7 days, i.e., at 21 and 28 DAS. The observations on disease severity were taken at 28 DAS. The disease severity of individual plants was scored by visual observation of the uprooted plant roots using the 1–9 scale (Nene et al., 1991) 28 DAS. The values from the above scale were then brought under a 1–4 modified scale for better distribution of the observations (Table 2). This modified scale was further used to derive percent disease susceptibility index (DSI) (Padaria et al., 2016a) of different treatments by using the following equation:

$$\mathrm{DSI}\left(\%\right)=\left[\left(4*A_{4}+3*B_{3}+2*C_{2}+1*D_{1}+0*E_{0}\right)/4N\right]*100$$

**TABLE 2:** Modified disease severity scale for dry root rot of chickpea.

Actual scale	Modified scale	Disease severity
1	0	No infection on roots
2–3	1	Very few small lesions on roots
4–5	2	Lesions on roots clear but small and new roots free from infection
6–7	3	More lesion on roots; many new roots generally free from lesions
8–9	4	Roots infected and completely discolored

[*A*_4_ denotes the number of plants recording the score 4, *B*_3_ the number of plants scoring 3, and so on; *N* denotes the total number of plants (*A*_4_ + *B*_3_ + *C*_2_ + *D*_1_ + *E*_0_) under the particular treatment.]

The whole plants were uprooted during each sampling with minimal to no damage to the roots, and adhered soil particles were removed by washing thoroughly using de-ionized water. The roots were blotted dry, harvested by cutting near the collar region using a sterile blade, and immediately flash-frozen using liquid nitrogen. The frozen roots were preserved at −80°C until further used.

### Quantification of *R. bataticola* Colonization

The frozen root samples were used for quantifying the *R. bataticola* colonization within the tissue. Total genomic DNA (gDNA) was extracted from the root samples and *Rb*6 isolate using the PureLink Plant Total DNA Purification Kit (Invitrogen, Untied States) according to the manufacturer’s protocol. Frozen sample of 100-mg amount was finely grounded and re-suspended in 250 μl suspension buffer provided in the kit and vortexed for complete homogenization. To the homogenized mixture, 15 μl each of 20% SDS and RNase A (20 mg/ml) were added, mixed well by inversion, and incubated at 55°C for 15 min. After precipitating proteins and polysaccharides using a precipitation buffer, the DNA clean-up was undertaken using PureLink^®^ Spin cartridge columns. Finally, the gDNA bound to the columns was eluted by adding 50 μl of elution buffer and stored at −20°C for downstream experiments. The quality and quantity of gDNA were measured using 0.8% agarose gel and NanoDrop spectrophotometer analysis (Multiskan Go NanoDrop Spectrophotometer, TS, Untied States).

The absolute quantification of *R. bataticola* DNA was performed using qPCR. The Rb-F3 and Rb-B3 primers (Table 3) were designed from the conserved region of partial ITS and 5.8S rRNA sequences of *R. bataticola*. qPCR was carried out in C1000 Touch^TM^ Thermal Cycler with CFX 96^TM^ Real-Time System (Bio-Rad, Untied States). The 20-μl reaction mixture consisted of 10 μl 2X KAPA SYBR Green PCR Master Mix (Kapa Biosystems, Untied States), 500 nM of each primer, and 10-fold diluted gDNA individually as template DNA. The PCR thermal cycling conditions were programmed as follows: 95°C for 3 min (initial denaturation) followed by 40 cycles of 95°C for 10 s (denaturation) and 62°C for 30 s (annealing and extension) at which the sensors detect fluorescence. Further, a melt curve was generated by measuring the continuous fluorescence at 60–90°C, where a 0.5°C temperature was increased per second. The threshold cycle (*C*_*t*_) values based on the detection of fluorescence were determined by the Bio-Rad software. The gDNA of isolate *Rb*6 was 10-fold serially diluted to obtain a range of DNA concentration from 10 ng/μl to 0.01 pg/μl. The *C*_*t*_ value for amplification was determined by qPCR following the above protocol. A standard curve was prepared by plotting Ct values against the log DNA concentration as per Sharma et al. (2015). The amplification efficiency (%) was calculated using online qPCR efficiency calculator software (Thermo Fisher Scientific), where the slope was derived from the standard curve.

**TABLE 3:** List of all primer sequences used for this study.

S. no.	Gene/region	Name	Primer sequence	Amplicon size
1	ITS	Rb-F3 (F)	CCTCCCACCCTTTGTATACCTACC	191
		Rb-B3 (R)	CGATGCCAGAACCAAGAGATCCG	
2	Actin (reference)	qCP Actin (F)	GTGGTGGTTCTACTATGTTCCC	115
		qCPActin (R)	CTGTATTTCCTCTCTGGTGGTG	
3	PR-2 (β-1,3-endoglucanase)	qCP β gluc (F)	GGTCGGCTACTTCGTATGATAAC	216
		qCP β gluc (R)	TCCTTCTTTCTCCACCAAATCC	
4	PR-3-type chitinase (*CHI* III*)*	qCP Chit III (F)	CTTGCAACACAAACAACTACCA	217
		qCP Chit III (R)	TCAGCGGAGTTCAGAGAGTA	
5	PR-5 (thaumatin-like)	qCP Thaumatin (F)	TCAGTTGCACAGCCGATATT	205
		qCP Thaumatin (R)	GTGCTAGTTGGGTCATCTTGAG	
6	PR-12 (defensin)	qCP Defensin (F)	TGGCTTGTGCTTCCTCTT	192
		qCP Defensin (R)	GTGCACCAACAACGAAAGTC	
7	Endochitinase	qCP EndoChi (F)	GTCCTTACCCTGATGCTCATTT	142
		qCP EndoChi (R)	GTCCATTGATTCCAAGCATTAACA	
8	*PAL* (phenylalanine ammonia-lyase)	qCP_PAL1 (F)	ACTCTTCCCGATCCACTCA	180
		qCP_PAL1 (R)	CTCGACACGAACACCACTATC	
9	*CHS* (chalcone synthase)	qCP CHS (F)	GAATACATGGCACCTTCATTGG	162
		qCP CHS (R)	AGGCATGTCAACACCACTT	
10	*FLV I* (flavonoid 3′-monooxygenase)	qCP Flav1 (F)	CAATGGACACTTCTGCAACATC	193
		qCP Flav1 (R)	GCCACAGGATGGAGTCTAAAG	
11	*PO* (peroxidase)	qCP Perox (F)	GTTCAGGGTTGTGATGGTTCTA	199
		qCP Perox (R)	TAACATCACGGGTTGCCATAG	
12	*LOX* (lipoxygenase)	qCP *LOX* (F)	TTAAGACATGGGTCCAAGAGTATG	204
		qCP *LOX* (R)	GAGCAGAAGCAGTCCATATGAT	
13	*LEA I* (late embryogenesis abundant gene)	qCP LEA I (F)	GTGAGACCATGGGCCGAAC	199
		qCP LEA I (R)	TTGGGCTGTCTGACTGGT	
14	*LEA II* (late embryogenesis abundant gene)	qCP LEA II (F)	AGGTGCAACTGATGCTGTGA	180
		qCP LEA II (R)	GCGTTGAATAAAAACCAAATTACGA	
15	*DREB* (dehydration-responsive element binding protein-2A)	qCP *DREB* (F)	AGCACATGTTAGTGAAAAGCCA	176
		qCP *DREB* (R)	CAAGGCGGGCGTTCAGTT	
16	*NECD* (9-cis-epoxycarotenoid dioxygenase gene)	qCP NECD (F)	ACCCACGTGTCCAAATCTCC	160
		qCP NECD (R)	CGGCTACCGGTTCGTAATGT	

*All primer sequences have been sourced from our previous publications; Ghosh et al. (2017) (s. no. 1) and Tarafdar et al. (2018) (s. no. 2–16).*

### Real-Time Quantitative Analysis of Gene Expression

The harvested root samples from different time-points were further used to isolate the total plant RNA using the UniPro RNA Isolation Kit-Plant (Dr. KPC Life Sciences, India) according to the manufacturer’s protocol. Root tissue samples of 30 mg amount were finely ground using 300 μl each of buffers URP1and Sol 1. The suspension was then incubated at 70°C for 15 min with frequent vortexing at 2-min interval. After further treatment using buffers URP2 and URP3, the RNA was finally eluted in 50 μl of nuclease-free water. The quality and quantity of the extracted RNA were confirmed using a 1% agarose gel and a NanoDrop spectrophotometer. The RNA was stored at −20°C for further downstream process.

The cDNA synthesis was undertaken using the SuperScript III cDNA synthesis kit (Invitrogen, Untied States) according to the manufacturer’s protocol. Each 20 μl of the reaction mixture contained 10 μl of 2X RT Reaction Mix, 2 μl of RT Enzyme Mix, and up to 1 μg of RNA. The contents were mixed well and incubated at 25°C for 10 min and later incubated at 50°C for 60 min. Here, the reaction was terminated at 85°C for 5 min and immediately chilled on ice. Finally, 1 μl (2 U) of E*scherichia coli* RNase H was added and incubated at 37°C for 20 min. The concentration of each cDNA was measured using a NanoDrop spectrophotometer and dilutions prepared accordingly. Such cDNA was used for studying the expression profiles of defense-related genes in chickpea during its interaction with *R. bataticola.* A total of 14 stress-responsive genes of chickpea were taken for expression profiling to have an insight into the host–pathogen interaction at the molecular level during the combined stress scenario (Tarafdar et al., 2018). These included 10 host defense-related genes further grouped into three categories: (i) PR genes, viz., PR-2 (β-1,3-endoglucanase), PR-3 type chitinase (*CHI* III), PR-5 (thaumatin-like), PR-12 (defensin), and endochitinase gene; (ii) genes related to phytoalexin biosynthesis, viz., chalcone synthase (*CHS*), flavonoid 3′ monooxygenase (*Flav* 1), and phenylalanine ammonia-lyase (*PAL* 1) gene; and (iii) genes related to reactive oxygen species (ROS) metabolism, viz., lipoxygenase (*LOX*) and peroxidase (*PO*) gene; and four moisture stress-responsive genes, viz., late embryogenesis-abundant genes (*LEA* 1 and *LEA* 2), 9-cis epoxy carotenoid dioxygenase gene (*NCED*) and dehydration-responsive element binding protein-2A (*DREB*-2A).

The actin gene of chickpea was used as the endogenous reference gene for normalizing the gene expression (Tarafdar et al., 2018), whereas cDNA of non-inoculated chickpea root tissues from 25°C and 80% SMC conditions at 14 DAS served as the experimental control to normalize and compare the gene expressions of plant defense-related genes. The qPCR reactions were carried out in a 20-μl reaction mixture as described in section “Real-Time Quantitative Analysis of Gene Expression.” The *C*_*t*_ values of three technical replicates from three biological replications (each biological replication comprised of pooled root tissues from three samples) were averaged to determine the expression profile of each gene, while the primer specificity was confirmed using the melting curve analysis. The 2^–ΔΔ*CT*^ method using *C*_*t*_ value (Livak and Schmittgen, 2001; Padaria et al., 2015, 2016b) was used to calculate the relative expression of the genes. The expression profiles of the genes are explained in terms of genotype, temperature, SMC, and time-points in the below results.

### Statistical Analysis

The statistical significance of the disease susceptibility, pathogen biomass colonization and difference in relative expression at different temperatures, SMC, and time points were calculated using three-way ANOVA in R-studio software. Data on percent disease susceptibility and the expression fold was subjected to arcsine transformation and log transformation, respectively, before the analysis. The residuals were subjected to Shapiro–Wilk normality test and pairwise mean comparison, and the *p*-value adjustment was conducted by Tukey’s honestly significant difference (HSD) test during *post hoc* analysis.

Five genes (viz., *CHI* III, endochitinase, *CHS*, *LOX*, and *DREB*-2A) were selected for box plot analysis under four different chickpea × *R. bataticola* pathosystems (viz., 25°C + 60% SMC, 25°C + 80% SMC, 35°C + 60% SMC, and 35°C + 80% SMC). The genes were selected based on their overall expression pattern and the subsequent interaction effects (Table 4) they produced in relation to the simulated environmental conditions. For each individual gene, the expression data (in fold) throughout the infection period, from both genotypes, were averaged together and plotted into box plots using MS Excel, displaying the distribution of data in minimum, first quartile, median, third quartile, and maximum expression values across the genes.

**TABLE 4:** Summary of interaction among different defense-related gene expression, disease susceptibility, and fungal biomass in chickpea (BG 212 and JG 11) during interaction with *R. bataticola* under different temperatures (25 and 35°C) and soil moisture conditions (60 and 80%).

Gene	Genotype	Summary of interaction Pr(>F)							Expression range (fold change)^†^	Average fold change^#^
				
		Temp	SMC	Time	Temp/SMC	Temp/time	SMC/time	Temp/SMC/time		
PR-2	BG 212	•	***	***	*	***	***	***	0.28–4.24	1.78
	JG 11	*	***	ns	ns	***	**	ns	0.24–1.49	0.72

PR-3	BG 212	***	***	***	ns	***	***	*	0.5–9.90	3.48
	JG 11	***	***	***	ns	***	ns	***	0.22–5.23	1.99

PR-5	BG 212	***	ns	*	ns	***	*	ns	1.08–6.48	3.20
	JG 11	**	***	***	ns	***	•	**	0.489–8.37	3.70

PR-12	BG 212	***	***	***	ns	***	***	*	0.13–6.87	2.31
	JG 11	***	**	***	ns	ns	ns	***	1.11–10.69	3.92

Endochitinase	BG 212	***	***	***	*	***	***	**	0.93–9.19	4.05
	JG 11	ns	***	***	*	***	***	***	0.93–5.35	3.16

PAL	BG 212	***	***	***	***	***	***	***	0.05–2.34	0.74
	JG 11	***	*	***	***	*	***	ns	0.05–2.40	0.55

CHS	BG 212	***	***	***	***	***	***	***	0.66–9.86	4.06
	JG 11	•	***	ns	***	***	*	*	0.19–2.22	0.89

FLV I	BG 212	***	***	***	ns	***	***	*	0.16–8.22	2.70
	JG 11	**	***	***	***	***	*	•	0.52–4.37	1.73

PO	BG 212	***	***	***	ns	***	**	ns	0.44–6.09	2.37
	JG 11	***	***	•	ns	***	ns	*	0.36–2.56	0.97

*LOX*	BG 212	***	***	***	***	***	***	***	0.43–7.99	3.40
	JG 11	***	***	***	***	***	ns	***	0.18–2.47	1.09

LEA I	BG 212	***	***	***	***	***	***	***	0.01–4.78	1.92
	JG 11	ns	***	**	***	***	**	***	0.48–5.05	2.70

LEA II	BG 212	***	***	ns	***	***	***	***	0.11–6.11	1.03
	JG 11	**	***	***	ns	**	ns	***	0.22–3.69	1.40

*DREB*	BG 212	***	***	***	***	**	***	***	1.58–16.59	7.14
	JG 11	***	***	*	***	***	**	***	0.57–20.15	7.51

NECD	BG 212	***	**	***	ns	**	***	***	0.32–5.54	1.94
	JG 11	ns	***	*	***	***	*	*	0.54–4.53	1.88

Disease susceptibility	BG 212	***	***	***	***	***	***	***	–	–
	JG 11	***	***	***	***	***	***	***	–	–

*R. bataticola* colonization	BG 212	***	ns	***	**	**	***	**	–	–
	JG 11	***	***	***	•	***	*	**	–	–

Significant codes: ***0.001; **0.01; *0.05; ^∙^0.1; ^ns^non-significant; –, not applicable. ^†^Range of minimum and maximum expression fold of the genes during experiment. ^#^Activity of the gene throughout the infection period (average gene expression of all time-points).

## Results

### Disease Susceptibility Index

**FIGURE 1:**
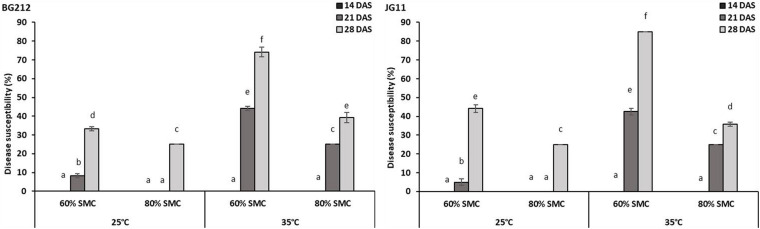
Disease susceptibility index of dry root rot (DRR)-infected chickpea roots at 28 days after sowing (DAS) under different abiotic stress conditions. *x*-axes show the temperature regime and SMC; *y*-axes show the percent level of disease susceptibility. Bars show the mean index values; error bars show the standard error calculated from three biological replicates. Means with different letters are significantly different [Tukey’s honestly significant difference (HSD), *p* < 0.05].

The factors temperature, SMC, and time-points were found to be statistically significant (*p* < 0.001) in influencing DSI in both BG 212 and JG 11. The interaction effects of the above factors also similarly proved significant (*p* < 0.001) for both (Table 4). As evident from the graphs (Figure 1 and Supplementary Figures 1, 2), time-point had the highest influence over the disease susceptibility. Irrespective of genotypes, the DSI was observed to gradually increase over time with no disease recorded up to 14 DAS and the highest disease susceptibility recorded at 28 DAS. During all time-points, we observed an overall low DSI at 80% SMC; however, DSI was significantly higher in all the time points at 60% SMC. Similarly, the indices from 35°C were much higher when compared to the counterparts at 25°C. A combination of 35°C with 60% SMC produced the highest DSI of 74.2 and 85% in BG 212 and JG 11, respectively, at 28 DAS.

### *Rhizoctonia bataticola* Colonization in Chickpea Root Tissue

**FIGURE 2:**
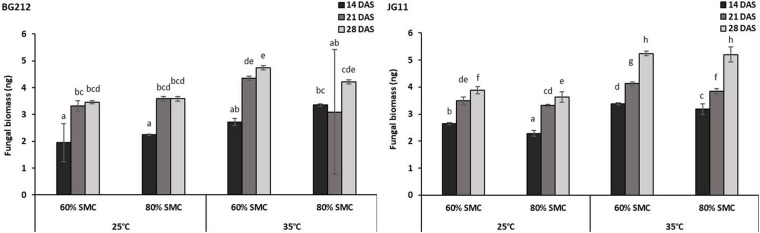
*Rhizoctonia bataticola* colonization in root tissues of chickpea genotypes BG 212 and JG 11. *x*-axes show the temperature regime and SMC; *y*-axes show the fungal biomass (nanogram) in chickpea root tissue. Error bars show the standard error calculated from three biological replicates. Means with different letters are significantly different (Tukey’s HSD, *p* < 0.05).

For a better understanding of the DRR disease progression in infected chickpea plants under different temperature and soil moistures, the colonization of *R. bataticola* in the root tissues was quantified using the real-time qPCR assay. For absolute quantification of *R. bataticola* DNA within chickpea root DNA, a standard curve was generated (Supplementary Figure 3). The slope of the linear regression curve and its correlation coefficient (*R*^2^) was observed to be −3.345 and 0.998, respectively. Based on this, the PCR efficiency was calculated to be 99.05%. The interaction effect of temperature, SMC, and time-point was significant (*p* < 0.01) in the colonization of the chickpea root tissues by *R. bataticola* (Table 4). The fungal biomass in root tissues of chickpea was recorded at 14 DAS. Colonization pattern was found different for both genotypes. The fungal biomass was observed to increase gradually throughout the time-points. In BG 212, the peak fungal biomass was observed at 21 DAS and remained non-significant from that observed at 28 DAS. The fungal colonization at 80% SMC was on par with those at 60% SMC throughout the time-points in JG 11 at both the temperatures, unlike in BG 212, where at 35°C, the fungal biomass was recorded to be slightly higher for 60% SMC than its 80% SMC counterpart. Also, throughout infection, the colonization was found to be significantly higher at 35°C, than at 25°C. Although the interaction effect of SMC is significant, the interaction results showed that temperature is more important than SMC for fungal colonization in BG 212 (*p* < 0.001) (Table 4). For both genotypes, the fungal biomass from 35°C was approximately 1.4-fold higher than that from 25°C, at 28 DAS (Figure 2).

### Differential Expression of Biotic and Abiotic Stress-Related Genes

#### Differential Expression of PR Genes

**FIGURE 3:**
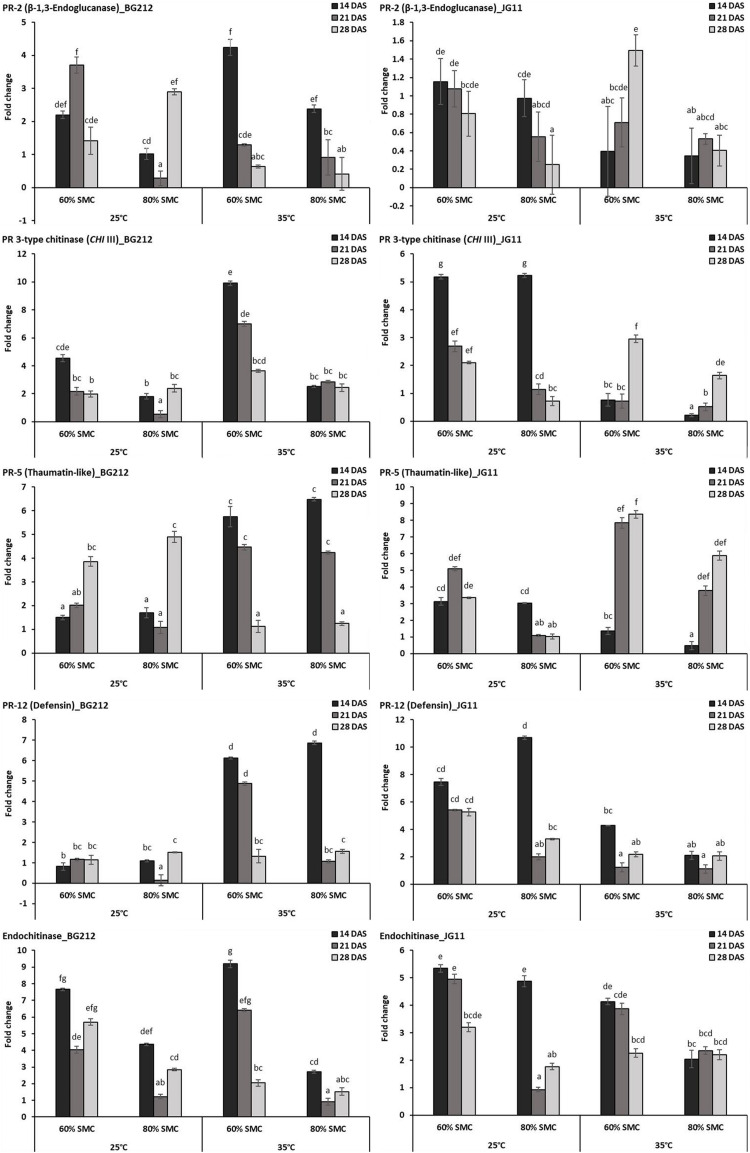
RT-qPCR analysis of differentially expressed pathogenesis-related (PR) genes in chickpea during interaction with *R. bataticola* under different temperatures (25 and 35°C) and soil moisture conditions (60 and 80%). Non-inoculated chickpea from 25°C and 80% SMC conditions at 14 DAS served as the experimental control. *x*-axes show the temperature regime and SMC; *y*-axes show the fold change in gene expression. Error bars show the standard error calculated from three biological replicates. Means with different letters are significantly different (Tukey’s HSD, *p* < 0.05).

Differential gene expression during chickpea × *R. bataticola* interaction was studied on both chickpea genotypes BG 212 and JG 11. Significant variations in the expression profile of different PR genes were observed through both up-regulation and down-regulation, between the genotypes, temperature gradient, as well as SMC. The factors temperature, SMC, and time-points influencing the PR gene expression in BG 212 were found to be statistically significant (Table 4). All the PR genes tested in BG 212 at 35°C were found to be highly up-regulated during initial periods of stress imposition. The genes encoding PR-2, *CHI* III, and endochitinase were highly up-regulated at 60% SMC than at 80% SMC, whereas the expression of PR-5 and PR-12 genes was on par with each other at both SMC, for the above temperature. In all cases, the maximum expression was observed in the early days after infection (14 DAS), which thereafter followed a decreasing trend. At 25°C, *CHI* III, PR-5, and PR-12 genes produced a low level of expression or were relatively down-regulated as compared to those at 35°C. The only exception was PR-5, where the gene was over-expressed in both 60 and 80% SMC up to 3.9- and 4.9-fold, respectively, at 28 DAS. PR-12 gene responsible for defensin protein produced the least expression of the above genes. PR-2 gene, producing β-1,3-endoglucanase enzyme, was also observed to be up-regulated up to 3.7-fold under 60% SMC at 21 DAS and up to 2.9-fold under 80% SMC at 28 DAS, while the gene encoding endochitinase was over-expressed at both initial and final stages up to 7.7- and 5.7-fold, respectively (Figure 3).

Except in PR-2, the interaction effect of temperature, SMC, and time-points on other PR gene expression (*CHI* III, PR-5, PR-12, and endochitinase) was found to be significant in the genotype JG 11 (Table 4). At 35°C, an overall low expression of all the PR genes was observed in JG 11 as compared to BG 212, with the only exception of PR-5, where the gene was found to be over-expressed at 21 and 28 DAS in both the SMC. For the PR-5 gene at 28 DAS, a maximum expression up to 8.4-fold was observed under 60% SMC, which was significantly higher than that under 80% SMC producing up to 5.9-fold expression. For the genes encoding PR-12 and endochitinase, the expression at 80% SMC was low but on par with each other. In the case of PR-12, a slight up-regulation in the expression was observed under 60% SMC at 14 DAS, whereas a higher expression was noted in the endochitinase gene at both 14 and 21 DAS for the same. At 25°C, PR genes produced an overall higher expression in JG 11 than those at 35°C. The expression trend of gene encoding PR-5 was similar to that of its 35°C counterpart, and the gene was up-regulated up to 5.1- and 3.4-fold at 21 and 28 DAS, respectively, under 60% SMC. *CHI* III and endochitinase gene followed a decreasing trend, where the maximum expression occurred at 14 DAS for both 60 and 80% SMC and was on par with each other. A similar trend was followed by the gene encoding PR-12, where over-expression occurred under both soil moistures at 14 DAS, but a maximum expression up to 10.7-fold occurred under 80% SMC compared to 7.4-fold under 60% SMC (Figure 3).

#### Differential Expression of Phytoalexin Biosynthesis Pathway Genes

**FIGURE 4:**
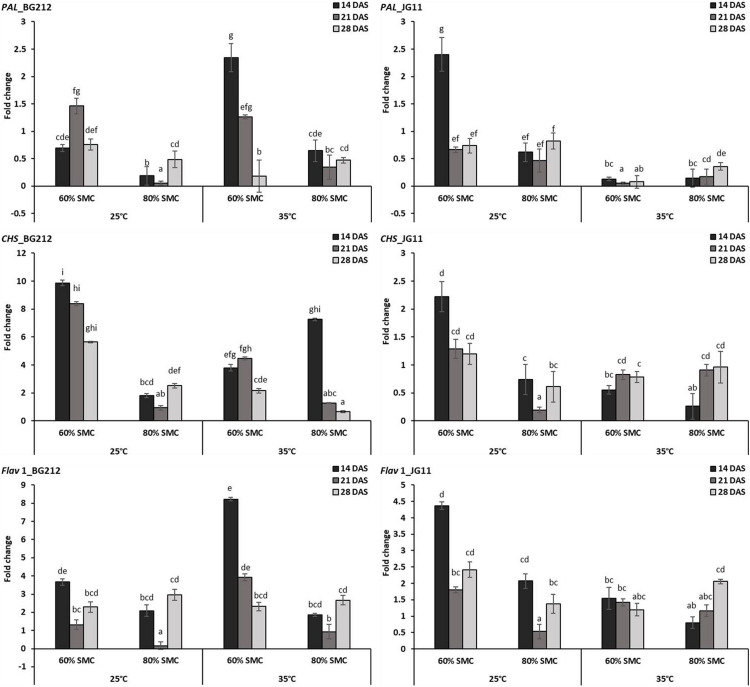
RT-qPCR analysis of differentially expressed phytoalexin biosynthesis pathway genes in chickpea during interaction with *R. bataticola* under different temperatures (25 and 35°C) and soil moisture conditions (60 and 80%). Non-inoculated chickpea from 25°C and 80% SMC conditions at 14 DAS served as the experimental control. *x*-axes show the temperature regime and SMC; *y*-axes show the fold change in gene expression. Error bars show the standard error calculated from three biological replicates. Means with different letters are significantly different (Tukey’s HSD, *p* < 0.05).

In BG 212, the expression of the *PAL*, *CHS*, and *Flav* 1 was significantly (*p* < 0.001) influenced by the temperature, SMC, time-points, and subsequent interactions. In JG 11, the factors time-point and temperature remained non-significant during the expression of CHS and Flav 1, respectively, but otherwise proved significant in the interaction effect (Table 4). At both temperatures, the expression of *CHS* was highly up-regulated for the genotype BG 212, at 25°C; the maximum expression up to 9.9-fold was realized for 60% SMC at 14 DAS and thereafter gradually decreased during the later course of infection. In the case of JG 11, the expression of *PAL* 1 gene was 2.4-fold higher under 60% SMC at 14 DAS. A similar trend, i.e., 2.3-fold expression, was observed for BG 212 in 60% SMC at 14 DAS, which, during further time points, was observed to decrease. *Flav* 1 gene at 25°C was fairly up-regulated in both genotypes during the initial and final time points, while at 21 DAS, the expression was observed to be steeply down-regulated before moving up. On the contrary, at 35°C, the maximum expression of *Flav* 1 gene up to 8.22-fold was observed in genotype BG 212 at 14 DAS under 60% SMC, thereafter being down-regulated (Figure 4).

#### Differential Expression of ROS Metabolism Pathway Genes

**FIGURE 5:**
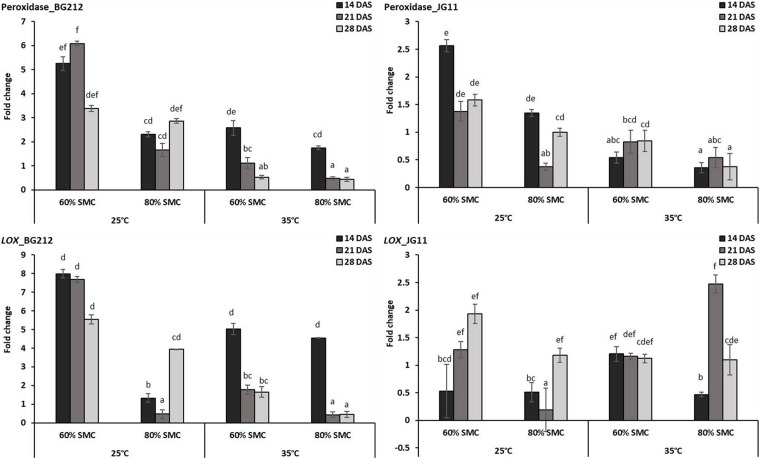
RT-qPCR analysis of differentially expressed reactive oxygen species (ROS) metabolism pathway genes in chickpea during interaction with *R. bataticola* under different temperatures (25 and 35°C) and soil moisture conditions (60 and 80%). Non-inoculated chickpea from 25°C and 80% SMC conditions at 14 DAS served as the experimental control. *x*-axes show the temperature regime and SMC; *y*-axes show the fold change in gene expression. Error bars show the standard error calculated from three biological replicates. Means with different letters are significantly different (Tukey’s HSD, *p* < 0.05).

In JG 11, the temperature, SMC, time-points, and their subsequent interactions produced significant changes in the gene expression of *PO* (*p* < 0.05) and *LOX* (*p* < 0.001) (Table 4). At 25°C, the genes coding for *PO* and lipoxygenase activity were found to be more up-regulated in both genotypes BG 212 and JG 11. In BG 212, expression of the above genes was high at 14 and 21 DAS under 60% SMC, but thereafter a steep down-regulation was recorded. At 25°C at 60% SMC, the genes encoding for *PO* produced the maximum expression up to 6.1-fold and *LOX* up to 8-fold in BG 212 at 21 and 14 DAS, respectively. In JG 11, the overall expression of both the genes was found to be fairly low as compared to BG 212. At 25°C at 60% SMC, the maximum expression of *PO* was up to 2.5-fold and *LOX* up to 1.9-fold in JG 11 at 14 and 28 DAS, respectively (Figure 5).

#### Differential Expression of Moisture Stress-Responsive Genes

**FIGURE 6:**
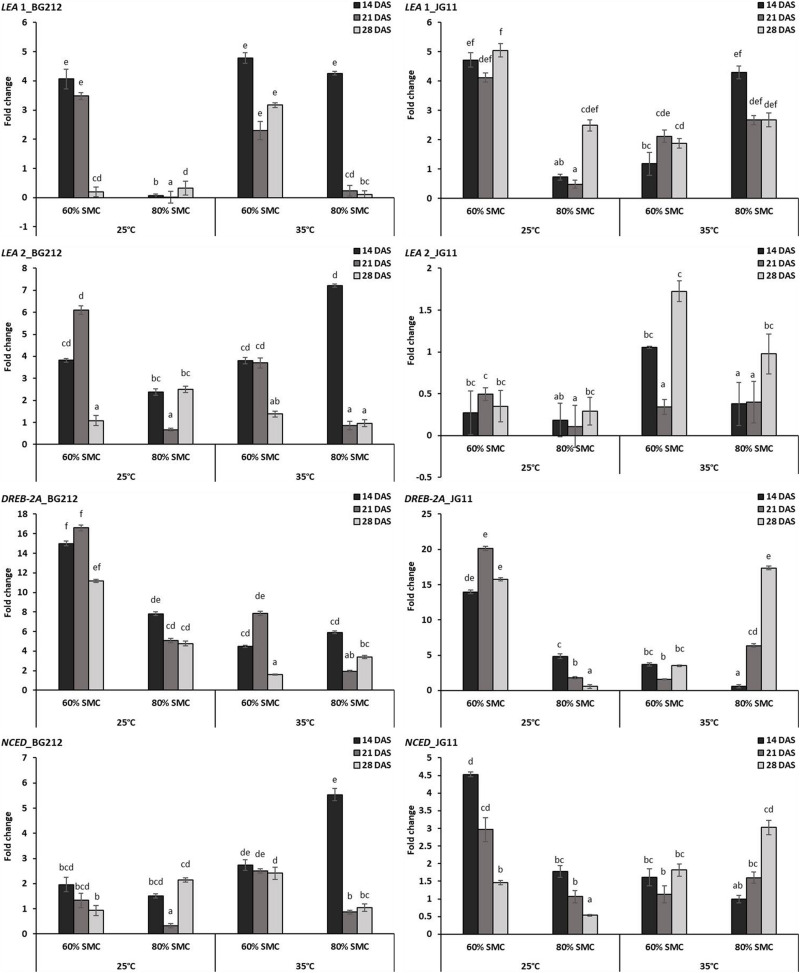
RT-qPCR analysis of differentially expressed moisture stress-responsive genes in chickpea during interaction with *R. bataticola* under different temperatures (25 and 35°C) and soil moisture conditions (60 and 80%). Non-inoculated chickpea from 25°C and 80% SMC conditions at 14 DAS served as the experimental control. *x*-axes show the temperature regime and SMC; *y*-axes show the fold change in gene expression. Error bars show the standard error calculated from three biological replicates. Means with different letters are significantly different (Tukey’s HSD, *p* < 0.05).

The interaction effect of temperature, SMC, and time-points on different moisture stress-responsive genes was found to be highly significant (*p* < 0.001) in both genotypes, but the interaction effect for *NCED* gene in the genotype JG 11 was only significant at 5% (Table 4). At 25°C, a very high expression for *LEA* 1 gene was observed at 60% SMC for both genotypes BG 212 and JG 11, while the expression at 80% SMC remained minimal. BG 212 at 35°C also followed a similar trend, whereas the expression of *LEA* 1 for JG 11 at 80% SMC was higher than 60% SMC. Here, an inverse trend was observed between the expressions of both SMC; the expression of *LEA* 1 at 60% SMC followed an increasing trend with time, whereas the expression of the gene at 80% SMC followed a declining trend over time. Also, the expression of *LEA* 2 was only significant for the genotype BG 212, while JG 11 under both temperature conditions showed very low expression at both SMC. At 25°C, the expression of BG 212 under 60% SMC gradually increased, reaching the maximum of up to 6.11-fold at 21 DAS, before declining. The gene encoding *DREB-*2A was found to be highly up-regulated at 60% SMC than at 80% SMC under 25°C. For the same, the maximum expression of up to 16.59- and 20.15-fold was realized for BG 212 and JG 11, respectively, at 21 DAS. At 35°C, the highest expression up to 17.37-fold was observed in JG 11 under 80% SMC at 28 DAS, while in BG 212, the maximum expression of up to 7.86-fold was realized for 60% SMC at 21 DAS. The expression of the *NCED* gene was up-regulated in JG 11 at 25°C while the same was true in BG 212 at 35°C. In JG 11, the maximum expression up to 4.53-fold was attained under 60% SMC at 14 DAS, which thereafter followed a declining trend. At 35°C, BG 212 showed the highest expression up to 5.54-fold under 80% SMC at 14 DAS, while the expression pattern of 60% SMC remained on par throughout all the time points (Figure 6).

#### Simulated Environment vs. Important Defense-Related Genes

**FIGURE 7:**
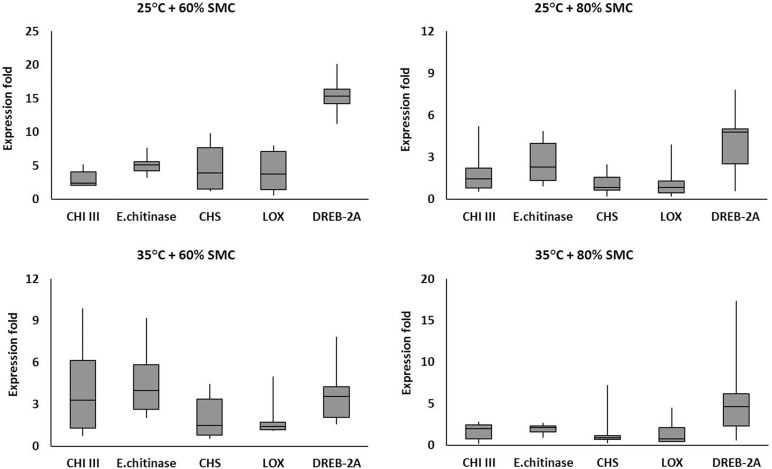
Comparative analysis of different defense-related gene expression in chickpea during interaction with *R. bataticola* under different temperatures (25 and 35°C) and soil moisture conditions (60 and 80%). The expression fold data of different defense-related genes throughout the infection period (14, 21, and 28 DAS) in two genotypes (BG 212 and JG 11) were averaged and plotted into box plots. *x*-axes show the selected genes; *y*-axes show the expression fold of the genes. The upper and lower side whiskers indicate the maximum and minimum expression values, respectively. Boxes in bars show the first quartile (25% percentile) and third quartile (75% percentile) across the genes. The line in between the boxes of each bar represents the median line (50% percentile).

The expression of the defense-related genes varied in diseased chickpea plants grown across the four different simulated environments (25°C + 60% SMC, 25°C + 80% SMC, 35°C + 60% SMC, and 35°C + 80% SMC) (Figure 7). The selected genes were highly expressed throughout the time period during infection. Irrespective of genotypes, the differential expression pattern and range of fold change were evident from the frequency distribution of the genes throughout the different simulated environment.

For example, the interaction effects of temperature, SMC, and time points influencing the endochitinase gene expression were highly significant for both genotypes, BG 212 (*p* < 0.01) and JG 11 (*p* < 0.001). Here in BG 212, the expression range of endochitinase was 0.93–9.19-fold with an average expression fold of 4.05 the entire infection period, while for JG 11, the expression range varied from 0.93- to 5.35-fold with an average expression fold of 3.16 (Figure 7). In another example, the main effects as well as the three-way interaction effects of temperature, SMC, and time points of the moisture stress-related gene *DREB*-2A were highly significant for both the genotypes (Table 4). Here, in BG 212, the expression range of *DREB*-2A was 1.58–16.59-fold with an average expression fold of 7.14 in the entire infection period, while for JG 11, the expression range varied from 0.57- to 20.15-fold with an average expression fold of 7.51 (Figure 7).

## Discussion

Plant diseases invariably respond to changes in climate scenario employing various synchronous interactions taking place among the host, pathogen, and environment (Garrett et al., 2016). Chickpea being mainly a rain-fed crop in India is grown in regions affected by varying intensities of soil moisture deficit due to the erratic rainfall and higher temperatures present in such ecologies (Gaur et al., 2008). Soil moisture being an acute climate variable has a great influence over various soil physiological and chemical parameters, thus affecting the growth of soil microorganisms such as fungi, bacteria, oomycetes, etc. (Rousk and Bååth, 2011). Plant pathogens with high multiplication rates and population dynamics may act positively under favorable weather conditions resulting in disease development (Agrios, 2005). *R. bataticola* is reportedly becoming intense in tropical-humid areas (Savary et al., 2011) but observed to be associated with low SMC and higher atmospheric and soil temperatures in chickpea. Although factors such as specific atmospheric heat, soil type, soil pH, type of amendments, cropping history, etc., are considered to influence DRR incidence either directly or indirectly, these may vary from crop to crop. However, soil moisture and atmospheric temperature was proven as the major factors for DRR predisposition in chickpea (Sharma and Pande, 2013; Sharma et al., 2015). A thorough understanding of such triggering factors is essential for the effective implementation of strategies for disease management. Since there is a current lack of quality information regarding chickpea × *R. bataticola* interaction, we have attempted to understand the molecular response of chickpea during DRR development under the influence of different SMC with varied temperatures.

In the present study, the combination of higher temperature (35°C) and low SMC (60%) was found to elicit the highest disease susceptibility in chickpea roots, while either low temperature (25°C) or higher SMC (80%) or a combination of both was found to lower the disease susceptibility and delay disease progression considerably. The increase in DSI over time can be translated to the gradual progression of disease in the root system. The above results corroborate with the findings of Sharma and Pande (2013), where the optimum temperature and SMC for DRR incidence in chickpea were found to be 35°C and 60%, respectively. Viana and Souza (2002) also reported 35°C to be the ideal temperature for growth and multiplication of *M. phaseolina*. Conserving the soil moisture reduced the *M. phaseolina* population in the soil, thereby reducing DRR incidence in cluster bean (Lodha, 1996). *M. phaseolina* showed higher growth, colonization, and survival rates in relatively dry soil conditions (Olaya and Abawi, 1996) as the viability of microsclerotia tends to rapidly decrease under high soil water levels (Shokes et al., 1977).

The study on *R. bataticola* colonization in chickpea root tissue revealed a time-course progression of the fungal biomass within the host root tissues. The fungal biomass was recorded slightly higher at 35°C as compared to 25°C but occurred irrespective of genotypes BG 212 and JG 11. Our studies also revealed that the *R. bataticola* colonization pattern of chickpea roots in 60% SMC did not vary significantly from that of 80% SMC irrespective of temperature and genotype, but contrary to fungal colonization, interestingly, the difference in DSI was significant. No or very low DSI was recorded in the roots and foliage during the initial time-points, although fungal colonization was recorded at 14 DAS, indicating the time taken by the plants to express the symptoms despite being colonized by the pathogen. The severity was found to be aggravated during the later stages of growth, which very well correlated with the fungal colonization. The results indicated that higher temperatures may prove encouraging for *R. bataticola* colonization, whereas the SMC majorly influences the DRR disease severity in chickpea. A higher atmospheric temperature invariably leads to elevated soil temperatures, which in turn means higher growth and survivability of infection propagules of *Macrophomina*. Landa et al. (2006) made a similar observation for another soil-borne chickpea disease and reported a shift in the resistance reaction of chickpea against *Fusarium oxysporum* f. sp. *ciceris*, where the plants showing moderate resistance to the pathogen under 24/21°C day/night temperature regime were converted to highly susceptible when the temperature was increased to 27/25°C. Kendig et al. (2000) states that the pathogen can invade and damage the plants under stressed conditions due to the weakened state of basal protection mechanism. Since drought stress is only gradually built-up, combined stress in the pathogen–low SMC combination scenario does not occur in a spontaneous manner (Ramegowda and Senthil-Kumar, 2015), which may explain the delayed disease severity expression in our study despite the higher colonization at early time-points.

The accretion of PR genes encoding β-1,3-glucanase and chitinase enzymes is one of the most studied and characterized plant defense responses (Linthorst and Van Loon, 1991). Both antifungal hydrolases are induced in coordination with other PR proteins in typical systemic-acquired resistance (Ryals et al., 1996). Our study also corroborates with the above findings as chickpea challenged with *R. bataticola* produced a pronounced activity of both PR-2 and PR-3 type chitinase, at different time-points under similar temperature and SMC. Tarafdar et al. (2018) similarly reported overexpression of PR-3-type gene in chickpea against *S. rolfsii* infection under soil moisture stress. An elevated PR-5 (thaumatin-like) and PR-12 (defensin) activity in chickpea against the DRR disease was also observed in our study, especially those under low SMC. It is reported that most of the plant defensin proteins show varying degrees of anti-fungal activity during infections incited by a broad range of phytopathogens (Thomma et al., 2002). Active lysis of the fungal membranes has been reported under high concentrations of thaumatin-like proteins, while lower concentrations affected the cell permeability, leading to increased uptake of other antifungal compounds (Velazhahan et al., 1999). Among all PR genes, the endochitinase gene showed a uniform higher expression at low SMC irrespective of temperature and genotype in our study. Broglie et al. (1991) reported that over expression of endochitinase gene in *Brassica napus* and *Nicotiana tabacum* resulted in lowering the disease symptoms due to infection by *Rhizoctonia solani*. Also, an increased expression of endochitinase gene during stress suggests the role of ethylene signaling pathway in the plant defense (Tarafdar et al., 2018). A higher expression of the above PR genes in both genotypes BG 212 and JG 11 during initial time-points could be related to the early colonization of the *R. bataticola* in the root tissues; successively, the gene expression profile is recorded to lower as the DSI increases and reaches maximum toward the final time-point. Also, in simulated environments of higher temperature (35°C) with low SMC (60%), the disease susceptibility and colonization of fungal biomass in chickpea were maximum. This could explain why the plant is eliciting an over expression of certain PR genes like *CHI* III and endochitinase as a mode of defending itself under combined stress circumstances.

Phenylalanine ammonia-lyase has an important regulatory role in the biosynthesis of secondary metabolites such as phytoalexins (Sarwar et al., 2001) and also acts as a positive regulator of salicylic acid-dependent defense signaling to combat phytopathogens through the phenylpropanoid metabolic pathway (Kim and Hwang, 2014). Daniel and Barz (1990) reported that the elicitation of chickpea cell suspension cultures leads to a substantial increase in the activity of *PAL* and *CHS*. *CHS* plays an important role in the flavonoid biosynthetic pathway and responds against both biotic and abiotic stress in plants. In our studies, the maximum activity of *CHS* and *Flav*-1 was found when the infected chickpea plants were under low temperature (25°C) and low soil moisture (60% SMC), especially during initial time-points. The successful colonization of *R. bataticola* in BG 212 and JG 11during the early stage of infection may have initiated a higher expression of such genes, thereby imparting resistance against the invading pathogen, thus explaining lower levels of DSI for the same. The overall activity of *PAL* was lower compared to the other phytoalexin biosynthesis pathway genes studied but still produced similar results as above. This may be since *PAL* is not the main regulatory or rate-limiting enzyme in the pathway as such but majorly involved in other pathways leading to the formation of cell wall-bound phenolics (Daniel and Barz, 1990).

Plants can respond to biotic stresses through hypersensitive reactions by involving dynamic changes in the ROS metabolism (Torres, 2010; Spoel and Loake, 2011; Cvetkovska and Vanlerberghe, 2013). It results in severe cellular damage and therefore regulated strictly through several enzymatic and non-enzymatic mechanisms (Tarafdar et al., 2018). *PO* and *LOX* involved in the antioxidant system against HR-inducing biotic stress take part in scavenging the harmful and redundant ROS (Naya et al., 2007). In our study, the *PO* and *LOX* activity was observed to be elevated in chickpea irrespective of genotypes under lower temperatures (25°C) and lower SMC (60% SMC), especially during the initial time-points of infection. This may suggest the lack of disease expression in the same despite the presence of *R. bataticola* in the root tissues of chickpea. At higher temperature, the activity of both genes encoding *PO* and lipoxygenase was drastically impaired leading to a higher degree of cell death and tissue maceration of chickpea roots during DRR disease development. We could ascertain that, at higher temperatures and lower SMC, the antioxidant system was inept in reducing the effects of any hypersensitive reaction during chickpea × *R. bataticola* interaction resulting from such treatments.

Late embryogenesis-abundant proteins are known to act as molecular chaperones to prevent the formation of damaging protein aggregates during water stress (Goyal et al., 2005). Olvera-Carrillo et al. (2010) reported that lack of *LEA* proteins may lead to susceptible phenotypes in mature plants recovering from severe dehydration. Similarly, *NCED* genes linked to ABA synthesis are expressed, resulting in the accumulation of ABA under conditions of drought or dehydration (Xiong and Zhu, 2003; Ye et al., 2012). The category under *DREB*s is involved in the expression of various stress-responsive genes in plants through participating in several ABA-independent stress tolerance pathways (Lata and Prasad, 2011). The *DREB-*2 promoters are reportedly induced by dehydration and high salt stress in transgenic *Arabidopsis* (Nakashima et al., 2000). In our study, all genes involved in the moisture stress-responsive category were showing higher activity under low SMC, with the expression of the *DREB-2*A gene to be several folds higher, as compared to others. Such genes were over-expressing in the initial course of DRR infection and later observed to be down-regulated once the disease has successfully been established. In simulated environments of the lower temperature (35°C) with lower SMC (60%), the disease susceptibility and colonization of fungal biomass in chickpea were comparatively lower. Here, genes such as *DREB-2*A, which is known for low-moisture stress responses, might also be involved in reducing the *R. bataticola* colonization and DRR susceptibility. Under the combined stress scenario of chickpea plants under low SMC, stress could be rendered susceptible to phytopathogens as well as indirectly influencing the pathways involved in several plant defense responses.

## Conclusion

The study clearly points toward the emerging threat due to DRR in chickpea and indicates higher temperatures and low soil moisture as key drivers for DRR expression in chickpea, especially in semi-arid tropics. High temperature renders chickpea plants susceptible to disease, whereas low SMC dictates the extent of rotting or severity of disease in the root system. The pathogen colonization of chickpea root tissues was pronounced at all the time-points despite the climatic conditions provided, suggesting the ability of *R. bataticola* to thrive and grow in a wide range of temperature and moisture conditions. The role of chemotaxis involving root exudates and border cells is yet to be investigated for the above scenario and could provide more insights into these findings. Substantial changes in the expression of defense-related genes occurred in chickpea in response to combined stress. Significant *in planta* over-expression was observed in genes coding for enzymes such as endochitinase and PR-3-type chitinase, in response to combined stress in chickpea plants. The expression can be observed to conform highly based on the overall simulated growth conditions and the time of exposure, but differed between the two genotypes. In the majority of cases, the over-expression of genes was found to be elicited under low SMC. The role of these genes in the chickpea defense system against *R. bataticola* could be further established using functional validation and deployed in resistance breeding programs; however, the challenge of understanding this concept in DRR-resistant genotype still needs to be studied. Also, the selected candidate genes in the above study do not represent all the stress-related genes. Understanding the role of genes related to stress hormones, cell wall rigidity, osmoregulation, etc., in the context of DRR is also important for better understanding of host–pathogen interaction and formulation of better management strategies. While an economically viable chemical control measure or highly resistant sources are yet to be achieved, our study signposts that life-saving irrigations especially toward the flowering and podding stages of the crop could arrest the DRR development and reduce disease severity to a great extent.

## Data Availability Statement

The original contributions presented in the study are included in the article/Supplementary Material, further inquiries can be directed to the corresponding author/s.

## Author Contributions

US in consultation with MS conceived, designed, and initiated the study. HM contributed in setting up the experiment. US and AT were responsible for analysis and interpretation of results and drafted the manuscript. MS provided critical inputs at various stages of the study and edited the manuscript. All authors have read and approved the manuscript.

## Conflict of Interest

The authors declare that the research was conducted in the absence of any commercial or financial relationships that could be construed as a potential conflict of interest.

## Supplementary Material

Supplementary Figure 1 DRR severity in chickpea roots (BG 212) vs. control at 28 DAS under different simulated abiotic stress conditions.

Supplementary Figure 2 DRR severity in chickpea roots (JG 11) vs. control at 28 DAS under different simulated abiotic stress conditions.

Supplementary Figure 3 Calibration of qPCR for *R. bataticola* quantification. Standard curve shows the correlation between the log10 DNA quantity (nanogram) vs. *C*_*t*_ values for 10-fold dilution of pure *R. bataticola* genomic DNA. *x*-axes show the DNA quantity (nanogram per microliter); *y*-axes show *C*_*t*_ values obtained from qPCR.
