# Supplementary figures and images for: Temperature and Soil Moisture Stress Modulate the Host Defense Response in Chickpea During Dry Root Rot Incidence
Source: Front Plant Sci. 2021 Jun 4;12:653265. doi: 10.3389/fpls.2021.653265 (PMC8213392; doi:10.3389/fpls.2021.653265)

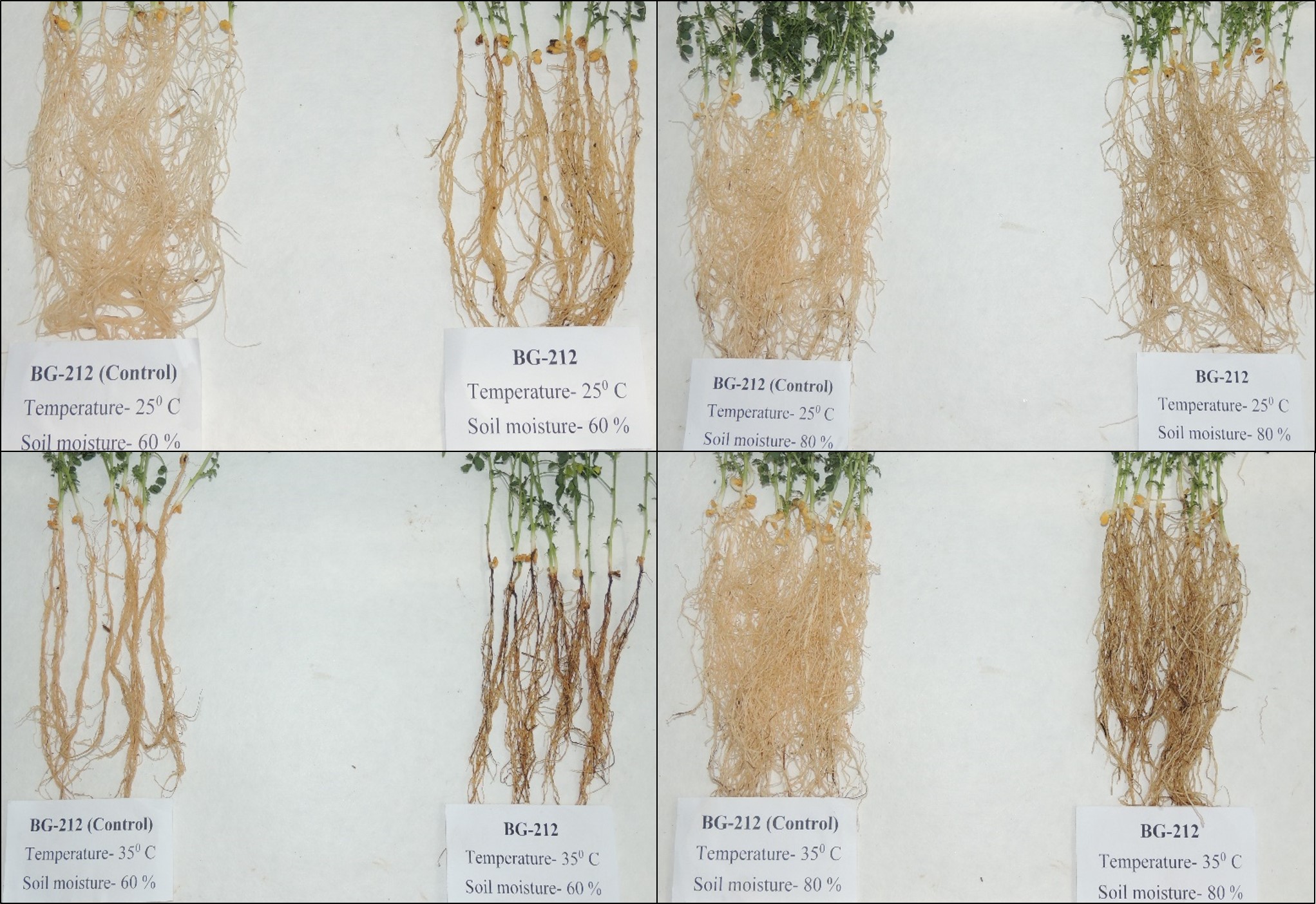

Supplement: Supplementary Figure 1 — DRR severity in chickpea roots (BG 212) vs. control at 28 DAS under different simulated abiotic stress conditions. [file Image_1.JPEG]

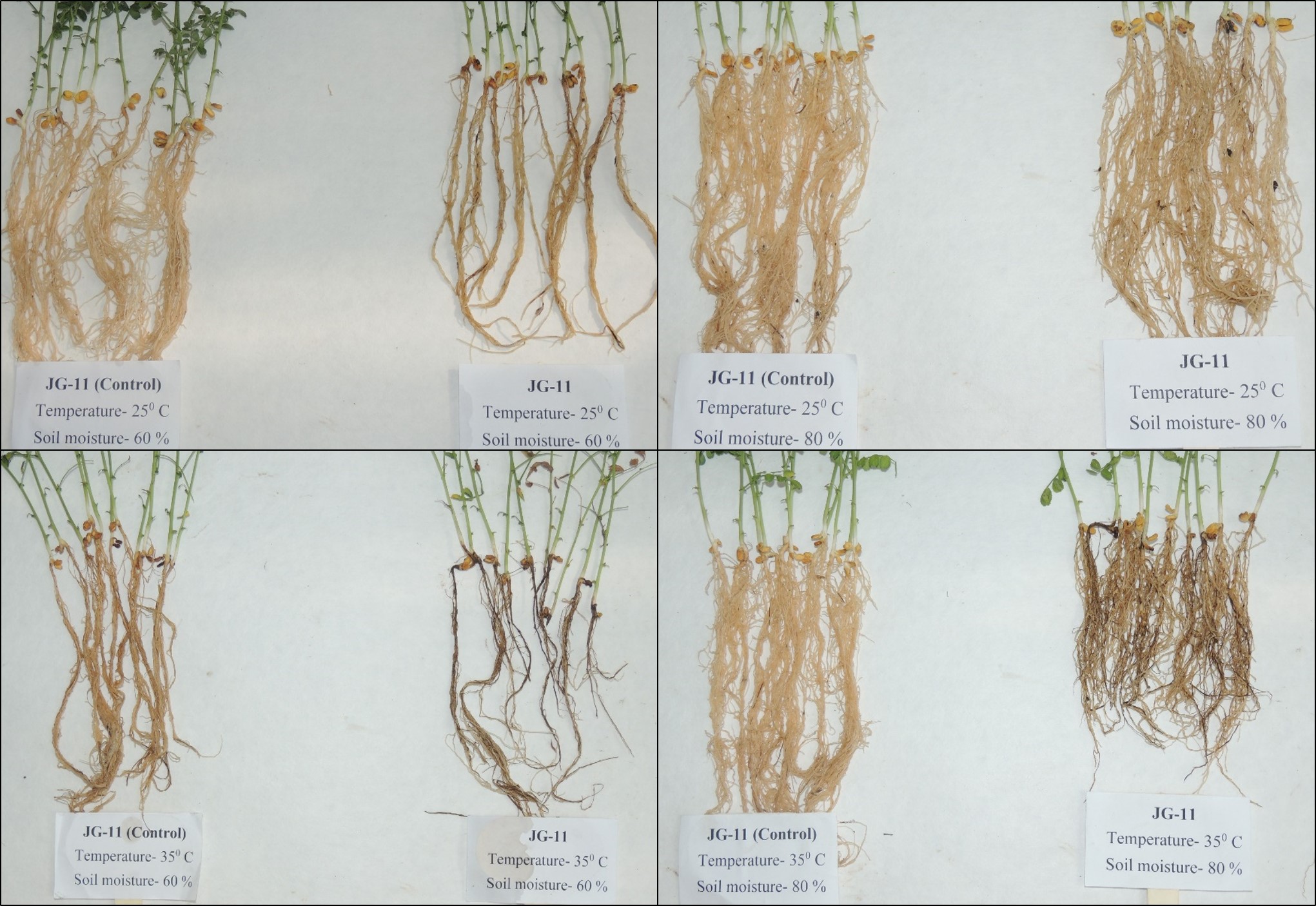

Supplement: Supplementary Figure 2 — DRR severity in chickpea roots (JG 11) vs. control at 28 DAS under different simulated abiotic stress conditions. [file Image_2.JPEG]

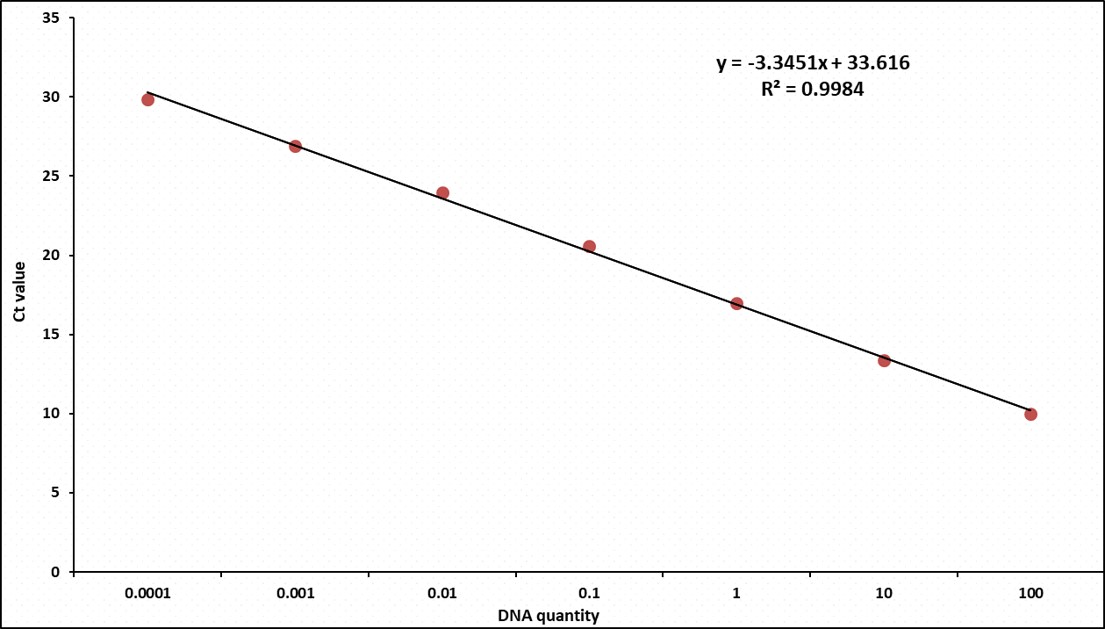

Supplement: Supplementary Figure 3 — Calibration of qPCR for R. bataticola quantification. Standard curve shows the correlation between the log10 DNA quantity (nanogram) vs. Ct values for 10-fold dilution of pure R. bataticola genomic DNA. x-axes show the DNA quantity (nanogram per microliter); y-axes show Ct values obtained from qPCR. [file Image_3.JPEG]
