# Supplementary material for: Temperature and Soil Moisture Stress Modulate the Host Defense Response in Chickpea During Dry Root Rot Incidence
Source: Front Plant Sci. 2021 Jun 4;12:653265. doi: 10.3389/fpls.2021.653265 (PMC8213392; doi:10.3389/fpls.2021.653265)
Supplement: Supplementary file 4 [file Table_1.DOCX]

**Table S1.** Gradients for temperature, relative humidity (RH) and light inside plant growth chambers (PGC)

| **Time** | **PGC 1** | | | **PGC 2** | | |
| --- | --- | --- | --- | --- | --- | --- |
|  | **Temperature (°C)** | **RH (%)** | **Light** | **Temperature (°C)** | **RH (%)** | **Light** |
| 00.00 | 22 | 60 | 0 | 12 | 60 | 0 |
| 06.00 | 25 | 60 | 3 | 18 | 60 | 3 |
| 10.00 | 30 | 50 | 5 | 22 | 60 | 5 |
| 12.00 | 35 | 50 | 7 | 25 | 60 | 7 |
| 15.00 | 30 | 50 | 5 | 22 | 60 | 5 |
| 18.00 | 26 | 60 | 0 | 18 | 60 | 0 |
| 23.59 | 22 | 60 | 0 | 12 | 60 | 0 |
